# Immersive Surgical Anatomy of the Retrosigmoid Approach

**DOI:** 10.7759/cureus.16068

**Published:** 2021-06-30

**Authors:** Roberto Rodriguez Rubio, Weipeng Xie, Vera Vigo, Anthony Lee, Ottavio S Tomasi, Ivan H El-Sayed, Adib Abla

**Affiliations:** 1 Neurological Surgery, University of California San Francisco, San Francisco, USA; 2 Neurosurgery, Paracelsus Medical University, Salzburg, AUT; 3 Otolaryngology, University of California San Francisco, San Francisco, USA

**Keywords:** retrosigmoid approach, surgical neuroanatomy, volumetric models, posterior fossa, cerebellopontine angle

## Abstract

The retrosigmoid approach (RS) approach is the workhorse of the posterolateral neurosurgical techniques to access various posterior fossa structures and even extends into the middle fossa. Many studies have detailed two-dimensional (2D) descriptions of the RS technique from either the lateral or posterior view. This study is the first to provide a comprehensive analysis of the RS technique, soft tissue, extracranial landmarks, and intracranial structures of the posterolateral region using interactive three-dimensional (3D) volumetric models (VMs). The visuospatial understanding of the neuroanatomical structures and landmarks of the RS approach is critical for successful surgeries with minimal complications. This study aims to create a collection of VMs and stereoscopic media for the relevant layer-by-layer soft tissue anatomy and step-by-step surgical technique of the RS approach using cadaveric dissections. Five embalmed heads and one dry skull were used to generate stereoscopic images and VMs using 3D scanning technology (i.e., photogrammetry and structured light scanning) to illustrate and simulate the RS approach. The extracranial structures were divided into myofascial, superficial vascular, superficial nerve, and bony anatomy. The RS approach was divided into seven major steps: patient positioning, incision of the skin, dissection of the scalp flap, dissection of the muscles, craniotomy, dural opening, and closure.

Additionally, we described an anatomical classification of surgical corridors based on the cisternal segments of the cranial nerves exposed during the RS approach. We discussed the nuances of the keyhole variations of the RS approach and intradural modifications of the RS approach using 3D VMs to illustrate the surgical corridors and the intradural structures accessed. These interactive VMs allow for clear visualization and dynamically immersive experience for neuroanatomical studies of the RS approach in 360-degrees and virtual reality (VR). Computer graphics can be implemented in neurosurgery to facilitate our topographic knowledge, which is crucial for anatomical education, surgical planning, intraoperative decision making, and postoperative care.

## Introduction

The unilateral approach to the cerebellopontine angle (CPA), the predecessor to the retrosigmoid (RS) approach, was first described in 1903 by Fedor Krause [1]. The RS approach was developed from the united efforts of neurosurgeons and neuro-otologists and has since been refined and modified upon by many surgeons to become the workhorse in neurosurgery for dealing with pathologies in the posterior fossa [2-4]. The RS approach provides a wide corridor of exposure for the CPA structures and a comparable working area and angle of attack to more aggressive approaches while minimizing bone removal [5,6]. Additionally, it may be extended inferiorly through the far-lateral transcondylar or far-lateral retrocondylar approaches or extended anteriorly to reach the middle fossa through intradural variations [4,5].

Extensive topographic and three-dimensional (3D) microanatomical studies of the RS approach are essential preoperative steps for a successful surgery. Most surgical descriptions and anatomical studies of the RS approach are limited to two-dimensional (2D) photographic representations from either the lateral or posterior angle; therefore, there is a need to combine the two views with present-day 3D technology to create interactive volumetric models (VMs) to facilitate our understanding of the layer-by-layer neuroanatomical structures of the posterolateral region of the skull. A comprehensive visuospatial understanding of the RS approach's soft tissue and bony structures is crucial for surgical planning, intraoperative decision-making, and postoperative care. The VMs in this article demonstrate the 3D representation of the soft tissue anatomy, surgical landmarks, and techniques of the RS approach serving as a valuable tool for anatomical education and structural understanding of the posterolateral skull base.

## Technical report

Materials and Methods

Five embalmed and latex-injected cadaveric heads were prepared for anatomical dissection and surgical simulation. One dried skull was utilized to identify and document the relevant anatomical structures of the RS approach. Dissections were performed under a surgical simulation setting using a surgical microscope (OPMI Pentero 900; Carl Zeiss AG, Germany). Stop-motion frames were recorded with a high-definition stereoscopic device (Truevision 3D; Truevision Systems, Goleta, CA). Stereoscopic (side-by-side) pictures were taken using a professional camera (D810; Nikon, Tokyo, Japan), and selected specimens were reconstructed using 3D data obtained via surface scanning techniques (i.e., photogrammetry and structured light scanning). Our lab had previously documented the comprehensive workflow using both techniques [7]. Additionally, anatomical base meshes of intradural structures were obtained from the BodyParts3D (The Database Center for Life Science, University of Tokyo, Tokyo, Japan). The VMs were post-processed and optimized for online use using computer graphics software (Zbrush 2021.5.1, Pixology Inc., California, USA; Nomad 1.49, Nomad Sculpt, © Stéphane Ginier and Blender 2.82, Blender Foundation, Amsterdam, Holland), and the corresponding texture maps were generated with a 3D texturing program (Substance Painter; Adobe, San Jose, CA, USA). No IRB/ethics committee approval was required for this study.

Virtual Platform

The anatomical VMs were uploaded to a web-based 3D model viewer (Sketchfab; Sketchfab Inc, New York, NY, USA), a platform that belongs to a series of new modalities meant to enhance the immersive and functional capacities of the VMs. Once the VMs were uploaded, the virtual scene was prepared for its real-time rendering. Position, lighting, materials, and filters were set to highlight the regions of anatomical interest. Strategic points were labelled and annotated for an interactive experience. Views of the models were set for both 2D and 3D experiences. Finally, the stereoscopic version of the virtual scene was set up and tested using a virtual reality (VR) headset (HTC Vive; HTC Co., Taiwan, China) and a browser compatible with WebVR technology (Firefox Nightly; Mozilla Co., Mountain View, CA, USA).

The following instructions can be used to manipulate all VMs: To move (left click and drag); To zoom in and out (use the mouse scroll). For smartphones and VR-ready computers (click on the glasses icon "view in VR"); To view annotations (click on the numbers); To move around the object (tap or press trigger on the floor using the blinking yellow circle as a pointer). For mobile augmented reality (AR), click on the AR icon (cube) in the top right corner and aim at a horizontal flat surface; when the surface is detected, tap on it to place the VM. To view the video in VR mode, Google Cardboard and YouTube mobile apps are needed. First, open the video on the YouTube mobile app and tap the Cardboard icon. Next, place the mobile device inside the Google Cardboard. Finally, look around to view the video in 360-degrees. The quality of the textures and navigation style can be modified by clicking on the gear "Settings" icon.

Indications

The RS approach can access structures located in the CPA, posterior fossa, internal auditory meatus (IAM), petroclival area, and lateral or anterior foramen magnum [4,5]. The RS approach also requires the least extensive soft tissue dissection and bony removal compared to other skull base approaches to the petroclival region, such as the combined petrosal and transcochlear approaches [6]. Therefore, the RS approach is a hearing-sparing technique applicable for pathologies such as microvascular decompressions, tumours (i.e., schwannomas, meningiomas, epidermoid cysts, arachnoid cysts, ependymomas, papillomas, metastases), and vascular lesions (i.e., cavernomas, aneurysms, arteriovenous malformations, dural arteriovenous fistulas); it can also be used for auditory brainstem or midbrain implants [4,5].

Anatomical Description

We will review the myofascial, superficial vascular, superficial nerve, and bony anatomy in the posterolateral region pertinent to the RS approach in the following sections. First, the soft tissues will be described layer-by-layer from superficial to deep, lateral to medial, and superior to inferior. Afterwards, the important bony landmarks for approximating the transverse sinus (TS), sigmoid sinus (SS), and transverse-sigmoid sinus junction (TSSJ) for the craniotomy will be discussed. A clear understanding of the soft tissue anatomy is necessary to avoid skin ischemia, muscle atrophy, dysesthesia, lesser occipital nerve injury, scar neuroma, or other postoperative complications following the RS approach [4,8]. The incision and dissection of the soft tissue should prioritize the preservation of extracranial vasculature, followed by extracranial sensory nerves, and lastly, the myofascial structures.

Myofascial Anatomy

Most blood vessels and nerves in this region are found at the level of the subcutaneous adipose tissue superficial to the musculatures described below. Superficially in the posterolateral region, the most lateral and superior muscle is the posterior auricular muscle (PAM). The PAM originates posteriorly from the mastoid of the temporal bone at the level of the superior nuchal line (SNL) and inserts anteriorly in the lower cranial surface of the concha, a hollow just posterior to the external auditory meatus (EAM). The PAM functions to pull the ear backward, a vestigial postauricular reflex that was responsible for orienting the pinna. On the same plane and inferior to the PAM is the mastoid portion of the sternocleidomastoid muscle (SCM) (Interactive model 1). The SCM inserts on the lateral surface of the mastoid at the lateral half of the SNL. Medial to the SCM, we find the trapezius muscle, which originates superiorly from the medial third of the SNL, the external occipital protuberance, and the nuchal ligament.

**Video 1:** Volumetric model of a specimen showing the superficial muscles, nerves, and arteries of the left posterolateral region.

**Figure 1:**
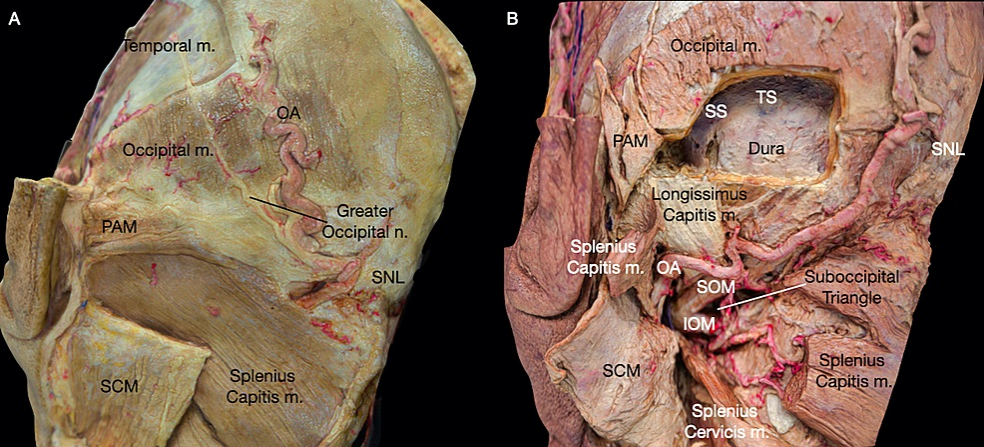
Overview of the myofascial and osseous anatomy of the left postauricular region. (A) Posterolateral view of the superficial myofascial layers. (B) Posterolateral view of the deep muscles and their relation with the retrosigmoid exposure. Greater occipital n.: greater occipital nerve; IOM: inferior oblique muscle; OA: occipital Artery; Occipital m.: occipital muscle; PAM: posterior auricular muscle; RS: retrosigmoid; SCM: sternocleidomastoid muscle; SNL: superior nuchal line; SOM: superior oblique muscle; Splenius capitis m: splenius capitis muscle; SS: sigmoid sinus; Temporal m.: temporal muscle; TS: transverse sinus.

Most laterally in the deep intrinsic muscles, the splenius capitis inserts on the lateral portion of the mastoid and the lateral part of the SNL lying underneath the mastoid portion of the SCM (Fig 1A). Underneath the splenius capitis, the longissimus capitis inserts at the posterior portion of the mastoid process. Medial to the splenius capitis and longissimus capitis, the semispinalis capitis inserts below the trapezius at the SNL just superior to the inferior nuchal line (INL) (Interactive model 1). Thus, the lateral insertion of the semispinalis capitis is partially covered by the medial insertion of the splenius capitis. In contrast, the medial insertion is covered by the superior portion of the trapezius.

In the deeper muscles of the neck, the posterior belly of the digastric muscle arises from the digastric groove on the inferior surface of the skull, medial to the mastoid process (Interactive model 2). The suboccipital muscles, which comprise the obliquus capitis superior, obliquus capitis inferior, rectus capitis posterior major, and rectus capitis posterior minor, lies underneath the semispinalis capitis (Fig 1B). Thus, two muscles are normally exposed in the RS approach: the obliquus capitis superior, which inserts at the INL under the lateral portion of the semispinalis capitis, and the rectus capitis posterior major, which inserts on the lateral half of the INL lying medial to the obliquus capitis superior and posterolateral to the condyloid canal.

**Video 2:** Volumetric model of a specimen showing the deep muscles of the left posterolateral region and the course of the occipital artery.

Superficial Vascular Anatomy

The posterior auricular artery (PAA), branching from the external carotid artery (ECA) posteriorly at the level of the angle of the mandible or even branching from the occipital artery (OA) [9], runs above the posterior belly of the digastric muscle (Interactive Model 1). The PAA then ascends posteriorly, emerging superficially between the auricle and mastoid tip (~1.2 cm posterior to the EAM [8]) and runs superiorly posterior to the auricle towards the vertex giving off three to five branches that perfuses the postauricular region and forms anastomoses with surrounding arteries (Interactive model 1). The auricular branch of the PAA perfuses the auricle and the PAM. The stylomastoid artery arises from the PAA and perfuses the styloid process, mastoid process, and facial nerve as it exits the stylomastoid foramen [9]. The occipital branch of the PAA runs backwards superficial to the SCM and anastomoses with the auricular branch of the OA. The PAA also forms anastomoses with the parietal branch of the superior temporal artery (STA) [8]. The PAA varies greatly in length, terminating at the level of the EAM or extending up to the vertex, and varies in its size and diameter, as large as or occasionally larger than the STA or the OA [10].

The OA, branching from the ECA inferior to the PAA, first runs below and then medially to the posterior belly of the digastric muscle [11]. The OA then ascends posteromedially and emerges from the occipital groove, medial to the digastric groove (Fig. 1B). It then runs in a superomedial fashion, superficial to the obliquus capitis superior and semispinalis capitis, covered by the SCM and splenius capitis towards the SNL, giving off ascending and descending branches (Interactive model 2) [8,11]. The superficial descending branch of the OA perfuses the muscles in the neck, while the deep descending branch of the OA anastomoses with the vertebral artery (VA) within the suboccipital triangle (Fig 1B) [11]. Finally, the ascending branch of the OA reaches the SNL, pierces through the fascia insertion of the splenius capitis, trapezius, and SCM at about 3.5 to 4 cm lateral to the inion [8], and runs tortuously towards the vertex branching extensively forming anastomoses with the STA and the PAA.

**Figure 2:**
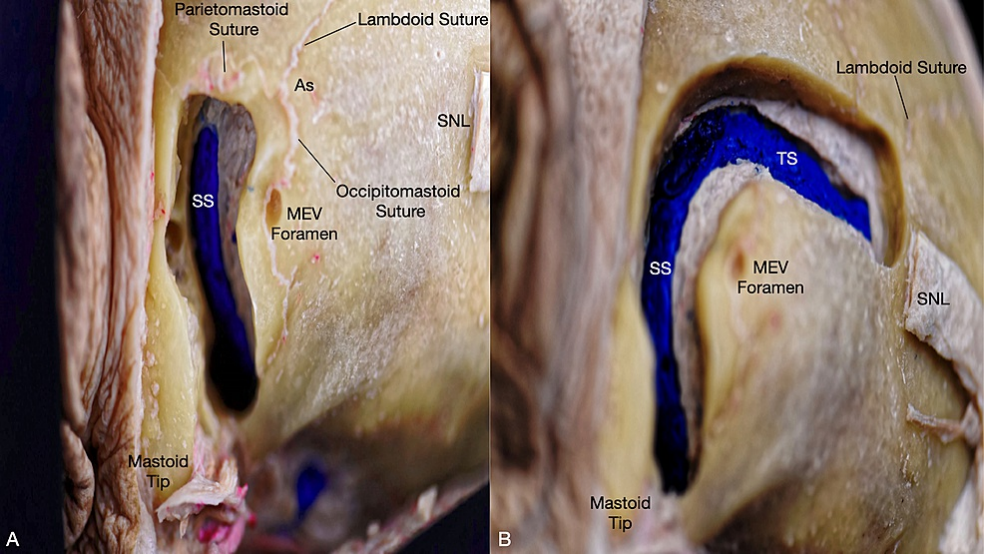
Posterolateral view of the left sigmoid and transverse sinuses and their relation to osseous landmarks. (A) Exposure of sigmoid sinus anterior and inferior to the asterion and its connection to the mastoid emissary vein. (B) Exposure of the transverse sinus running underneath the superior nuchal line. As: asterion; MEV: mastoid emissary vein; SNL: superior nuchal line; SS: sigmoid sinus; TS: transverse sinus.

The venous drainage of the posterolateral region is primarily carried out by the posterior auricular vein (PAV), occipital vein (OV), mastoid emissary vein (MEV), and the occipital emissary vein (OEV). The PAV arises from the plexus with the OV and superficial temporal veins. It runs down the mastoid process posterior to the auricle and drains into the external jugular vein. The OV arises from the posterior plexus of the vertex, converges into a single vessel which runs inferiorly piercing through the fascia insertion of the trapezius and SCM, and drains into the suboccipital plexus, vertebral plexus, deep cervical vein, external jugular vein, or the internal jugular veins [8]. The MEV is particularly important for the RS approach, as it is often cauterized or filled with bone wax to prevent significant bleeding or air embolisms [2,3,12]. The MEV allows venous drainage from the SS intracranially to the PAV and OV extracranially through the mastoid emissary foramen (Figure 2), which is usually found in the temporal bone near the occipitomastoid suture; however, the number of the mastoid emissary foramina is highly variable, ranging from zero to four [12]. The OEV is less prevalent than the MEV. However, when present, it usually forms a confluence with the MEV [12] and allows venous drainage from the TS through the occipital emissary foramen, which is usually found near the median nuchal line of the occipital squamous. The emissary veins serve as the primary venous outflow in the upright position when compared to the prone position. Thus they may serve as operative risks for the RS approach when performed in the sitting position [12].

Superficial Nerve Anatomy

The sensory nerves most important for the RS approach in the retroauricular region are the great auricular nerve (GAN), which serves the inferior half, and the lesser occipital nerve (LON), which serves the superior half. The GAN originates from the ventral rami of C2 and C3 of the cervical plexus, wraps around the posterior border of the SCM emerging at the Erb's point, and ascends superficially across the SCM towards the parotid gland. The GAN then arborizes into the anterior branch, which innervates the skin covering the parotid gland and anteroinferior auricle; the posterior branch, which innervates skin covering the mastoid process and the posterior auricle; and the lobular branch, which innervates the lobule. The LON originates from the ventral ramus of C2, sometimes also C3, of the cervical plexus, curves lateral to cranial nerve (CN) XI, emerging above Erb's point superior to the LON ascends superficially along the posterior border of the SCM (Interactive model 1). After piercing the deep cervical fascia near the cranium, the LON emerges posterior and superior to the GAN, arborizing in a fan-like fashion into the anterior branch, which anastomoses with the posterior branch of the GAN anteriorly, and the posterior branch, which anastomoses with the greater occipital nerve medially. The posterior branch of the GAN and the LON nerve are the most vulnerable during dissection, and their course is highly variable, so care must be taken to prevent damage to these nerves [8].

Bony Anatomy

An accurate understanding of the bony anatomy and natural boundaries is essential for identifying important landmarks to successfully access the CPA through the corridor of exposure provided by the RS approach. The exposure is limited anterolaterally by the EAM and SS, superiorly by the SNL and TS, inferiorly by the craniocervical junction, and posteromedially by the median nuchal line.

**Figure 3:**
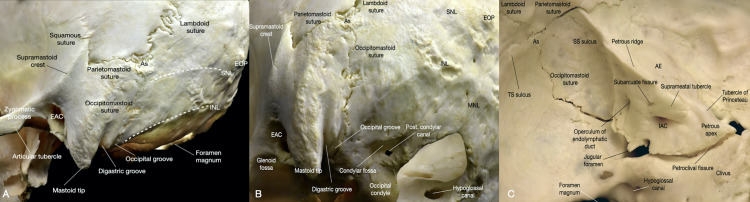
Overview of the bony anatomy relevant to the retrosigmoid approach. (A) Left lateral view of the skull. (B) Left posteroinferior view of the skull. (C) Internal view of the left posterior fossa and petrous face. As: asterion; AE: arcuate eminence; EAC: external acoustic meatus; EOP: external occipital protuberance; IAC: internal acoustic canal; INL inferior nuchal line; MNL: median nuchal line; Post. condylar canal: posterior condylar canal; SNL: superior nuchal line; SS: sigmoid sinus; TS: transverse sinus.

The asterion is one of the most crucial landmarks to identify in the RS approach because it is often used to estimate the TSSJ before the craniotomy begins (Figure 3). The name asterion derives from Greek and means "star/starry," additionally, it was also the proper name of the Cretan Minotaur. In anatomy is defined by the three-suture junction of the lambdoid, occipitomastoid, and parietomastoid sutures where the occipital, temporal, and parietal bones meet. Its reliability as a superficial landmark for the TSSJ for surgical approaches to the posterior fossa can vary in different patients [13]. The asterion can be located superficial, inferior, or superior to the TSSJ, SS, or TS. It is observed that the asterion is located superficial to the lateral half of the TS, directly superficial to the TSSJ, inferior to the TSSJ in the lateral posterior fossa, and superior to the TSSJ in 63%, 1%, 22%, and 14% of the time, respectively [13]. In general, from the asterion, the lateral TS is located directly underneath. The TSSJ is ~1 cm anteriorly, the SS is in the anteroinferior direction, and the posterior fossa dura is in the posteroinferior direction. While the asterion may not be the optimal bony anatomy to determine the TSSJ, other superficial anatomical landmarks can also be used for estimating the TSSJ, SS, and TS in the RS approach.

The zygoma-inion line, roughly the same as the projection of the SNL, can be used to locate the TS, the line between the mastoid tip and the asterion, or the vertical projection of the digastric groove, can be used to approximate the SS. Finally, the intersection between the zygoma-inion line and the mastoid tip-asterion line can be used for estimating the TSSJ.

Surgical Technique

Patient Positioning

The RS approach may be carried out with the patient in the supine, lateral, park-bench, sitting, or semi-sitting position. While every surgeon may have different preferences according to the specific case, this article will describe the operation in the semilateral position. The patient is positioned supine on the operating table with the operative side up. The ipsilateral shoulder is bolstered to rotate the patient's torso into a semilateral position, and the patient should be well secured onto the operating table. The head is rotated about 70-80 degrees to the contralateral side, flexed slightly anteriorly and towards the floor to open the angle between the occiput and the neck, and secured in place with a Mayfield clamp pin fixation. The pin placement should start after a scalp block and avoid superficial arteries (i.e., the STA, OA, and supraorbital artery) and superficial nerves. The placement should also avoid any thin bones prone to forceful pinning or bones covering the sinuses, such as the temporal squamous, inion, and areas over the TS or SS. For the RS approach, the double-pin arm is placed just superior and posterior to the contralateral superior nuchal line avoiding the suboccipital muscles, and the single-pin arm is placed just above the ipsilateral superior temporal line behind the hairline and anterior to the EAM, avoiding the forehead [5]. The ipsilateral shoulder may also be taped down to open the angle further and increase the workspace [2]. The mastoid tip should be the highest point in the surgical field. A lumbar drain may be placed at this time to facilitate cerebrospinal fluid (CSF) drainage in patients with moderate to large tumours [5,14].

Incision of the Skin

The common variation of skin incisions for the RS approach are the linear, lazy S-shaped, C-shaped, and curvilinear inverted U-shaped incisions (Interactive model 3). The linear and lazy S-shaped incisions are more simple and versatile, but the use of retractors result in the bunching of the skin and muscles and risk injuring the OA and occipital nerves; the C-shaped incision allows for a multilayer dissection and retraction of the skin flap and muscle flap which reduces postoperative CSF leak, but the inferior part of the incision may risk injuring the neurovascular trunks; the inverted U-shaped incision based inferiorly may preserve these neurovascular trunks, but it is performed in a single layer and cannot be extended superiorly [15]. Therefore, the C-shaped incision, popular among skull-base surgeons, will be described here.

**Video 3:** Volumetric model of a specimen showing the superficial landmarks to plan different skin incision variations for a right retrosigmoid approach.

After using the zygoma-inion line and mastoid tip-asterion line to approximate the TS, SS, and TSSJ, a C-shaped incision is drawn from 2 cm superior to the middle of the pinna to 1 cm medial to the mastoid tip with the apex of the curve 5 cm posterior to the postauricular crease. The incision may be extended inferiorly depending on the case. The surgical area is then prepped, draped, and lidocaine 1% with epinephrine is applied to the area. The skin is incised over the region of the temporalis muscle. It continues towards the mastoid tip making sure to cut through the subcutaneous adipose tissue, which may decrease the risk of GAN and LON damage. Special attention needs to be paid to the superior half of the incision to visualize and preserve the LON because it is more superficial and enters the region at the posterior edge of the subcutaneous tissue (approximately at the 4 o'clock position for the left side and at the 8 o'clock position for the right side). In case of an anteroinferior extension of the incision, care should be taken to avoid the risk of injury to the GAN at McKinney's point, which represents the superficial location of the nerve crossing the midransverse belly of the SCM at about 6.5cm inferior to the caudal edge of the EAM [8].

Dissection of the Scalp Flap

The subgaleal skin flap dissection is first carried out with scissors, then electrocautery inferior to the SNL through the subcutaneous tissue and fascia while keeping the thickness of the flap the same throughout. The skin flap is then elevated with the fascia of the PAM and the SCM and retracted anteriorly over the mastoid process.

Dissection of the Muscles

Traditionally, muscle dissection in the RS approach has been described as following the skin incision. The underlying muscles can be incised in line with the skin to create a single myocutaneous flap and retracted anteriorly in a C-shaped skin incision (Interactive model 4) [2], divided in line with the skin and retracted anteriorly and posteriorly in a slightly curved skin incision [5], or a single myocutaneous flap based along the suboccipital muscles can be reflected inferiorly in an inverted U-shaped skin incision [15]. Here, we describe an anatomical-based muscle dissection according to their respective insertion.

**Video 4:** Volumetric model of a specimen showing the muscular layer after incision and reflection of the skin for a right retrosigmoid approach.

A vertical fascial and periosteal incision is made from the mastoid tip inferiorly to the superior base of the skin flap superiorly over the mastoid process. The periosteum with the soft tissues is then dissected until the depression of the EAM and reflected anteriorly to expose the mastoid process. A horizontal incision along with the SNL and the PAM coupled with a small inferior releasing incision posteriorly is made to create a superior triangular fascia flap (i.e., occipitalis flap) over SNL and an inferior triangular fascia flap (i.e., SCM flap) below the SNL. The occipitalis flap is dissected and elevated over the occipital bone in an anterior-inferior to posterior-superior fashion and retracted using scalp hooks or sutured to the drapes; the latter is preferred [8]. The SCM flap is dissected and disinserted with electrocautery in an anterior-superior to posterior-inferior fashion until the horizontal crest of the suboccipital bone; then, the flap is retracted to expose the suboccipital bone, asterion, and emissary veins. The emissary vein(s) should be cauterized or gently obliterated using a local hemostatic agent to control the bleeding and prevent air embolisms. It is vital to bear in mind that overpacking the emissary vein(s) with hemostatic agents might cause sinus thrombosis.

Craniotomy

Following the retraction of the soft tissues, a craniectomy or craniotomy may be performed. The amount of burr hole depends on the patient's underlying pathology and the patient's age. Typically, more burr holes or a craniectomy should be employed for patients older than 60 years old because the dura is more attached to the cranial surface. The craniotomy may be limited to just the edge of the TS and SS in the standard RS approach or further extended to expose the sinuses with a limited posterior mastoidectomy in the extended approach (Interactive model 5).

**Video 5:** Volumetric model of a specimen showing the exposure of osseous landmarks during a right retrosigmoid approach after dissection and reflection of the muscular layer.

In the standard RS approach, the most important burr hole, which should expose the junction of posterior fossa dura and the margins of the adjacent TS and SS [13], is conventionally placed over the asterion or slightly below it [5,14]; however, it is also argued that the Teranishi technique (placing the burr hole 6.5mm inferior and 6.5mm anterior to asterion) or the Ribas technique (10 mm anterior to asterion with the superior edge of burr hole adjacent to the parietomastoid suture) are the most suitable approach for the placement of the initial burr hole in the standard and extended RS approach, respectively (Interactive model 5)[13]. The craniotomy is continued to expose the TSSJ, the inferior edge of the TS superiorly, the posterior edge of the SS anteriorly, and the floor of the posterior fossa inferiorly but not into the foramen magnum [4,8]. The initial cuts are first performed using footplate from anterior-superior to posterior-superior along the length of the TS, then from posterior-superior to posterior-inferior over the cerebellar hemisphere, and finally from posterior-inferior to anterior-inferior just behind the SS (Video 1). Care must be taken when approaching the most inferior portion of the occipitomastoid suture that leads towards the occipital groove, where the trunk of the OA lies.

The extended RS approach is preferred for the visualization and the skeletonization of the sinuses. A limited posterior mastoidectomy can be performed before or after the craniotomy to expose the entire SS [2,3]. Usually, the mastoidectomy is performed after the craniotomy, which provides a clear exposure of the cerebellar dura. The dural plane can be used to reference the dura covering the sinus (Interactive model 6).

**Video 6:** Volumetric model of a specimen showing the exposure of posterior fossa dura and sinuses after an extended right retrosigmoid approach.

**Figure 4:**
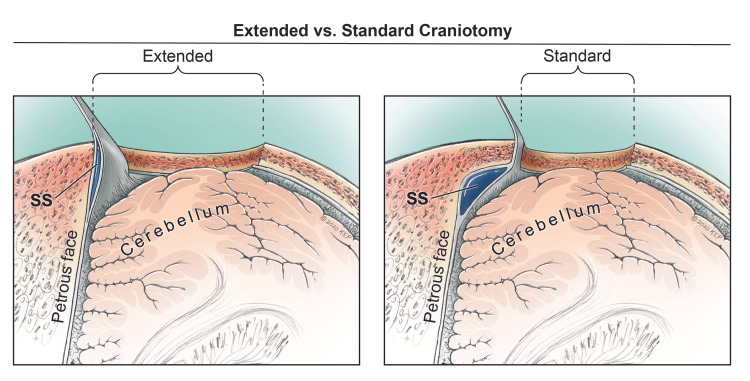
Right superior axial view showing the corridors for the extended and standard retrosigmoid (RS) approaches. The extended RS involves the skeletonization, visualization, and retraction of the sigmoid sinus (SS) and transverse sinus (TS). The standard RS is limited to the edges of the SS and TS. A wider surgical area and a wider attack angle between the cerebellum and petrous face can be achieved using the extended RS approach. SS: sigmoid sinus, TS: transverse sinus, RS: retrosigmoid approach.

When the mastoidectomy is performed before the craniotomy, the bone covering the SS is first thinned to eggshell thickness using a high-speed drill until blue colouring is visible, followed by bone removal using a diamond bit to expose the entire SS from the TSSJ to the jugular bulb. The inferior edge of the TS and the posterior margin of the surgical field over the cerebellar hemisphere is then exposed using a footplate [2]. It is vital to bear in mind that the dominant TS is about 7.5 mm, and the SS is about 8.5 mm in diameter, respectively. Therefore, drilling approximately 10 mm beyond the sinuses' visible edges is sufficient to uncover the sinuses thoroughly (Fig 4).

 

Alternatively, an extended RS craniotomy with skeletonization of both the TS and SS can be performed before the limited posterior mastoidectomy to expose the jugular termination of the SS (Fig 4) [3]. Four initial burr holes can be placed slightly superior to the medial part of the TS, slightly superior to the TSSJ, near the jugular termination of the SS, and over the cerebellar hemisphere to skeletonize both the TS and SS. The cuts are performed in the same directions as described above. The SS is the last to be skeletonized. The exposure of its entry into the jugular foramen requires a limited posterior mastoidectomy due to the angle of the mastoid process and the variable thickness of the compacted bone [3]. Further drilling inferiorly but superior to the occipital groove may increase the surgical work area according to the procedure.

When performing the craniotomy, it is preferable to preserve the shape of the craniotomy by starting and completing the cut from the outermost edge of the burr hole to keep the perimeter of the craniotomy as flush as possible. Any exposed mastoid air cells are sealed with bone wax or fibrin glue [4,5] to prevent postoperative CSF leakage.

Dural Opening

The posterior fossa dura is visualized under magnification with surgical loupes or an operating microscope. The exposed dura is predominantly irrigated by the meningeal branches of the VA and OA, and in some cases where the regional vasculature is pathological, proximal exposure of the trunks should be considered before opening the dura. The dural opening can be done with a medially based C-shaped incision along the inferior edge of the TS and the posterior edge of the SS 3-5 mm away, leaving the sinuses covered, which will allow subsequent watertight dural closure and prevents dura desiccation [4]. The surgical angle can be further increased by retracting the dura covering the sinuses after the extended RS approach [5]. However, any dural bleeding that requires cauterization and small dural tears will lead to dural shrinkage and difficulty in a watertight dural closing. Alternatively, the dura can be opened with a Y-shaped incision creating dural leaves based on the TS and SS (Interactive model 7) [14], or an X-shaped incision creating four triangular dural leaves laterally based on the SS, superiorly based on the TS, medially based, and inferiorly based.

**Video 7:** Volumetric model of a specimen showing the Y-shaped dural opening after a left retrosigmoid exposure.

**Figure 5:**
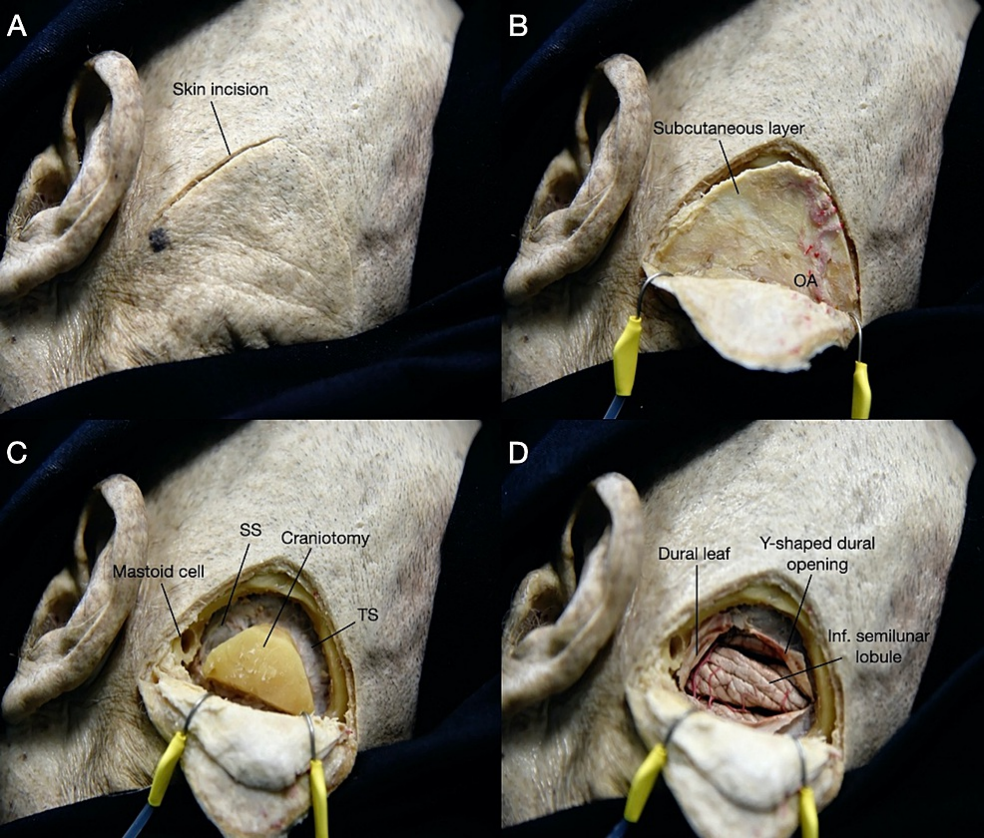
Left posterolateral view depicting a step-by-step dissection of the retrosigmoid (RS) approach using an inverted U-shaped incision. (A) After estimating the location of the sigmoid sinus (SS) and transverse sinus (TS), an inverted U-shaped incision is made. (B) The myofascial layer is dissected separately or reflected as a single myocutaneous flap. (C) The RS craniotomy is performed, exposing the edges of the SS and TS. (D) The dura is opened in a Y-shaped fashion exposing the posterolateral surface of the cerebellum. OA: occipital artery; SS: sigmoid sinus; TS: transverse sinus.

The reflection of these triangular dural leaves mobilizes the SS anteriorly and the TS superiorly, allowing for the maximum exposure of the CPA using the minimum number of cuts. The Y and X-shaped dural opening also help with dural closing even if dura cauterization is needed. The arachnoid is then cut, if necessary, to further drain the CSF from the cisterna magna to relax the cerebellum. Subsequently, the cerebellum should be protected from trauma using dynamic retraction with or without protective barriers (i.e., a surgical glove or cottonoid) to expose the CPA structures. The CPA microdissection is then performed according to the underlying pathology (Fig 5).

Closure

Before dural closure, careful inspection of the CPA and hemostasis should be achieved. The dura is then closed in a watertight fashion and reinforced with xenograft, tissue sealant, or dural patch if necessary. The bone flap is then replaced and secured using titanium plate and screws, or cranioplasty may be performed using methyl methacrylate or hydroxyapatite to decrease risks of CSF leaks and the formation of pseudomeningocele [8]. Finally, the muscle and the skin flaps are sutured back according to their respective anatomical layers.

## Discussion

Intradural Anatomy - The Lateral Triangles of the Posterior Fossa

**Figure 6:**
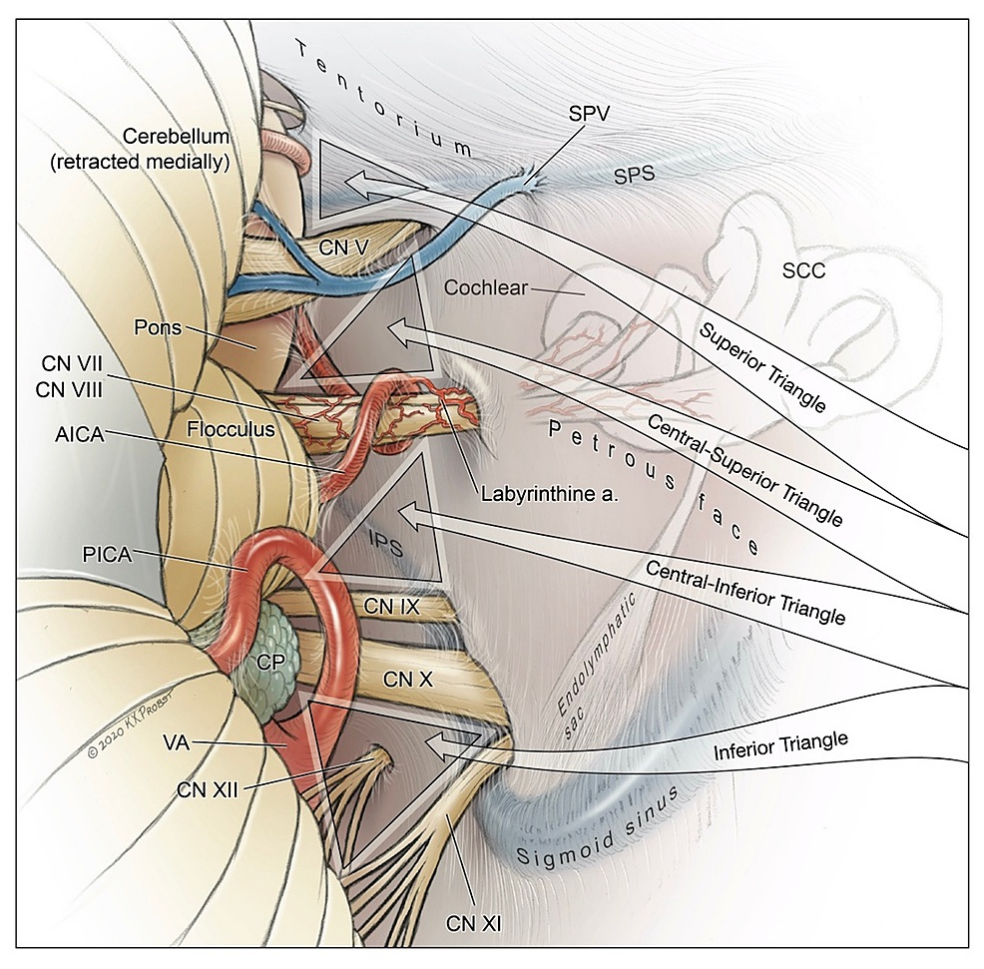
Illustration depicting a posterior view of the right lateral triangles of the posterior fossa and related neurovascular structures. AICA: anterior inferior cerebellar artery; CN: cranial nerve; CP: choroid plexus; CPA: cerebellopontine angle; IPS: inferior petrosal sinus; Labyrinthine a.: labyrinthine artery; PICA: posterior inferior cerebellar artery; SCC: semicircular canals; SPS: superior petrosal sinus; SPV: superior petrosal veins; VA: vertebral artery

The cisternal segments of the cranial nerves in the lateral posterior fossa form four virtual triangular corridors that can be used to access various peritruncal structures. These triangles are the superior triangle, central-superior triangle, central-inferior triangle, and inferior triangle of the lateral posterior fossa. The boundaries of each triangle are described in Table 1 and can be visualized in Fig. 6. It is important to bear in mind that patient positioning is crucial to clear visualization and reach for the anterior anatomical structures through these corridors.

**Table 1:** Summary of the lateral triangles of the posterior fossa and the anatomical structures accessed by the different keyhole RS variations. AICA: anterior inferior cerebellar artery; CN: cranial nerve; CP: choroid plexus; CPA: cerebellopontine angle; IAM: internal acoustic meatus; IPS: inferior petrosal sinus; IPV: inferior petrosal vein; LA: labyrinthine artery; PICA: posterior inferior cerebellar artery; SAA: subarcuate artery; SCA: superior cerebellar artery; SCC: semicircular canals; SPS: superior petrosal sinus; SPVs: superior petrosal veins; VA: vertebral artery

Superior Keyhole	Central Keyhole		Inferior Keyhole
Superior Triangle	Central-Superior Triangle	Central-Inferior Triangle	Inferior Triangle
Boundaries formed by			
-Tentorium -CN V -Lateral upper pons -Porus trigeminus	-CN V -CN VII/VIII complex -Middle peduncle -Suprameatal tubercle	-CN VII/VIII complex -CN IX -Flocculus -Posteroinferior surface of the petrous face	-CN X -CN XI -Rhomboid lip -Jugular tubercle
Structures accessed			
-CN V -CN VI -SCA -SPS -SPVs -Lateral pons -Tentorium -Porus trigeminus -Petrous apex -Dorello’s canal -Posterior cavernous sinus -Lateral upper and middle clivus	-CN V -CN VI -CN VII -CN VIII -AICA -LA -SAA -SPVs -Lateral pons -Middle peduncle -Trigeminal ganglion -Porus trigeminus -Suprameatal tubercle -IAM -Lateral middle clivus	-CN VII -CN VIII -CN IX -AICA -PICA -IPS -IPVs -Lateral pontomedullary junction -Flocculus -Bochdalek’s flower basket -Foramen of Luschka -IAM -Lateral middle and lower clivus	CN IX, CN X, CN XI, CN XII -PICA -VA -Lateral medulla -Olive -Rhomboid lip -Lateral lower clivus

Extracranial Variations - The Keyhole Retrosigmoid Approach

**Figure 7:**
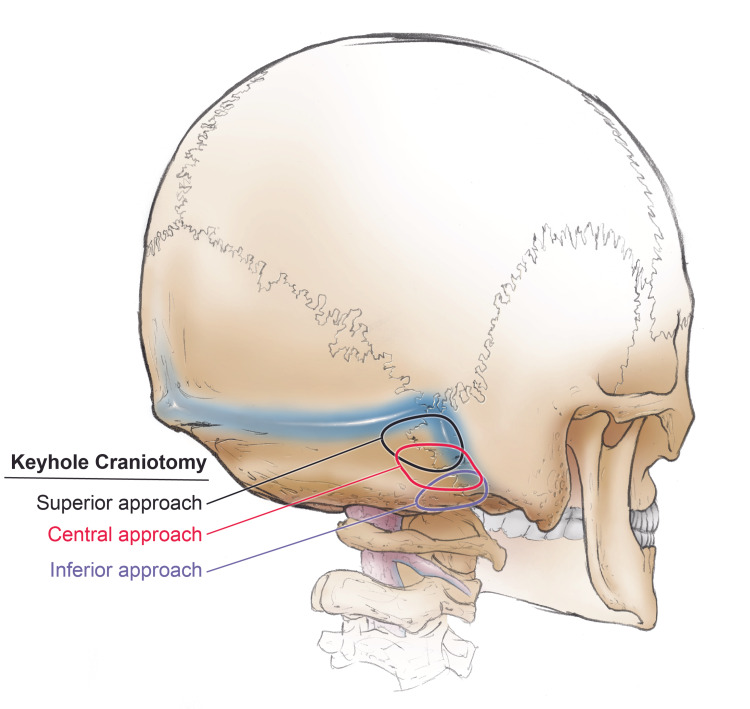
Depiction of right superior, central, and inferior variations of the keyhole RS craniotomy.

The keyhole RS approach is a minimally invasive procedure for specific pathologies that uses a limited craniotomy to reduce brain exploration and retraction to minimize damage to the intracranial structures and reduce postoperative complications [1,16]. This approach can be done microsurgically, purely endoscopically, or endoscope-assisted to improve illumination, viewing angle, and image quality for close-up positions [1]. The keyhole RS approach is usually employed for microvascular decompressions, intracranial dermoid cyst, cystic tumours, or small vestibular schwannomas [16]. The size, shape, and placement of the craniotomy need to be tailored to the specific target region and its underlying pathology. The superior variation is used for the upper neurovascular complex, the central variation for the middle of the CPA, and the inferior variation for the lower neurovascular complex (Fig 7) (Table 1).

The patient is positioned similarly to the RS approach, usually in the semilateral position with the head rotated toward the contralateral side. The head is also laterally flexed to different degrees according to the exact target in the keyhole variations [1]. After approximating the TS, SS, and TSSJ using anatomical landmarks, a 10 to 20mm craniotomy placement is outlined according to each variation, and the skin is incised. The skin may be incised horizontally parallel with the zygoma-inion line [16] or vertically over the posterior third of the planned craniotomy about 30 to 50mm in length [1]. The skin and the subcutaneous tissue are bilaterally retracted, revealing the SCM. A straight incision is performed longitudinally along with the SCM and retracted carefully anteriorly and more forcibly posteriorly to expose the occipitomastoid junction after freeing the myofascial attachment. Typically, a triangular craniotomy is placed at the inner curvature of the TSSJ for the superior variation, a quadrangular craniotomy below the TS and medial to the middle of the SS is used for the middle variation, and a triangular craniotomy at the inferior segment of the SS with an anterior and inferior border flush over the SS and the jugular bulb is performed for the inferior variation (Interactive Model 8). After a T-shaped or V-shaped dural opening, the CSF is drained from the cisterns and the intradural CPA dissection proceeds. After the procedure, the dura is closed in a watertight fashion. A cranioplasty is done with bone cement, and the soft tissues are sutured back after achieving adequate hemostasis. 

**Video 8:** Volumetric model of a skull with relevant venous structures and cranial nerves. The superior, central (superior and inferior), and inferior triangles are shown on the right side of the model. The shape of the three different keyhole approaches is shown on the left side of the model.

Intradural Variations - Retrosigmoid Intradural Suprameatal Approach and Posterior Intradural Petrous Apicectomy

**Figure 8:**
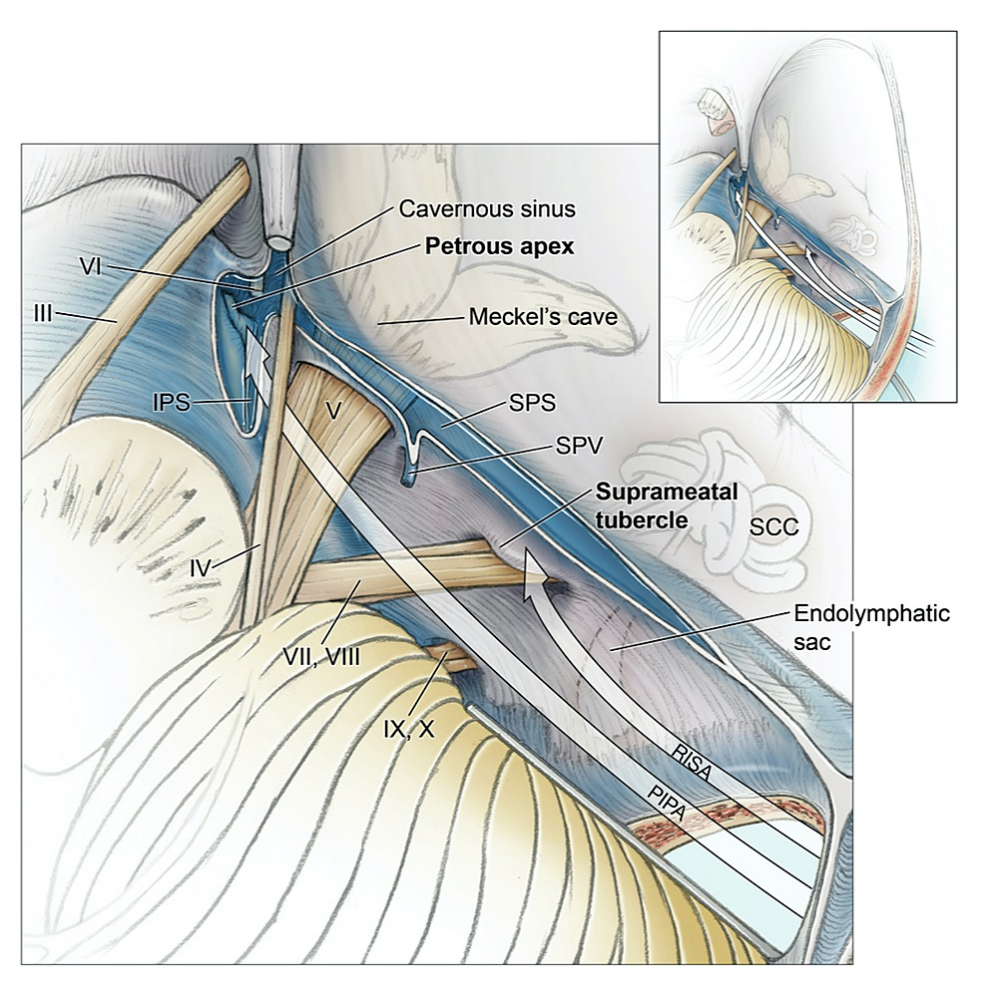
Superior view of the right posterior fossa and the surgical corridors to reach and remove the suprameatal tubercle and petrous apex. CN: cranial nerve; IPS: inferior petrosal sinus; PIPA: posterior intradural petrous apicectomy; RISA: retrosigmoid intradural suprameatal approach; SCC: semicircular canals; SPS: superior petrosal sinus; SPV: superior petrosal vein

The retrosigmoid intradural suprameatal approach (RISA) is an anterior extension of the RS approach to access tumoral lesions that extend to the Meckel's cave, petroclival junction, and posterior cavernous sinus through the removal of the suprameatal tubercle (Fig 8) [4,5]. The RISA can be done endoscope-assisted further to visualize the anteromedial middle fossa [5]. The RISA can be extended to properly access the anteromedial middle fossa through a posterior intradural petrous apicectomy (PIPA) by removing the petrous apex (Fig 8). The PIPA can be done endoscope-assisted to access the middle fossa, CN III, basilar trunk, and anterolateral brainstem [17]. The RISA is adequate for pathologies limited to the most anterior border of the posterior fossa, and the PIPA is needed for pathologies in the posterior fossa with minor extension into the posterior third of the cavernous sinus in the middle fossa [17,18]. The RISA combined with the PIPA can be employed for meningiomas, trigeminal schwannomas, epidermoid cysts, chondromas, chondrosarcomas, dura-invading chordomas, cholesteatomas, and lesions in the petroclival and petrous apex region [5,17,18]. The surgical corridor of the RISA and PIPA approach allows for safe access to the middle and anterior segment of the posterior fossa from the CPA, optimal visualization of the ipsilateral cerebellomedullary cistern, CN IV to XII, and AICA, and potentially lowering the risk of facial palsy, hearing loss, and damage to the labyrinth [17,18].

**Figure 9:**
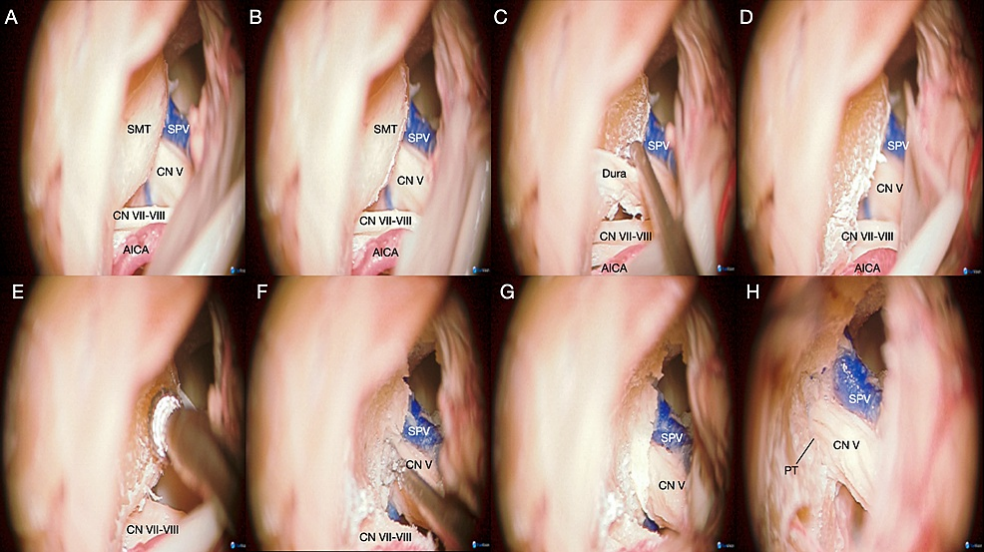
Microsurgical view of a left retrosigmoid intradural suprameatal approach (RISA). The step-by-step removal of the suprameatal tubercle during the RISA aims to improve the surgical freedom and surgical view anteromedially. (A) Identification of the main neurovascular structures surrounding the suprameatal tubercle (SMT). (B) Dural incision over the SMT. (C) Careful blunt dissection of the dura covering the SMT. (D) Complete exposure of the SMT. Ligation of the subarcuate artery might be needed to achieve proper exposure of the tubercle. (E) Drilling of the SMT. (F) Identification of the posteroinferior edge of the porus trigeminus (PT). (G) Exposure of the dura of the PT. (H) Opening of the posteroinferior portion of the PT, further medial drilling can be done in order to expose the ventral face of the trigeminal ganglion. AICA: anterior inferior cerebellar artery; CN: cranial nerve; PT: porus trigeminus; RISA: retrosigmoid intradural suprameatal approach; SCA: superior cerebellar artery; SMT: suprameatal tubercle; SPV: superior petrosal vein.

In the RISA approach, the CPA exposure steps are the same as the RS approach described above. The CPA portion of the tumour is first removed around the CN VII/VIII nerve bundles to expose and identify the intracranial structures within the CPA. The structure to identify and remove to gain access more anteriorly within the posterior fossa is the suprameatal tubercle (Fig 8). The size of the suprameatal tubercle is highly variable and may or may not obstruct the surgical view. While less prominent suprameatal tubercle does not limit the surgical view, it limits the surgical freedom for the manipulation of the surgical instruments. After the CN VII/VIII complex is protected with a piece of a rubber glove, the dura over the suprameatal tubercle is incised and resected. The suprameatal tubercle is removed towards the petrous apex anteriorly using diamond burrs to expose the CN V, upper clivus, and Meckel's cave (Fig 9) [5,18]. The degree of bone removal depends on the individual anatomy and pathology; however, one should bear in mind that the extension of the drilling is limited posterolaterally by the subarcuate fossa, the posterior and superior semicircular canals, and their common crus; inferiorly by the IAM and the CN VII/VIII complex; superiorly by the tentorium, and SPS; and anteriorly by the porus trigeminus (PT), CN V, and superior petrosal vein (Fig 7, 8). The CN V is carefully mobilized using smooth movements. The free edge of the tentorium may be opened parallel to the SPS, and the superior petrosal vein may have to be sacrificed to increase the surgical work area to allow for access to structures anteriorly [4,5]. After the suprameatal tubercle is removed, the PT can be properly visualized. The posterior wall of the cavernous sinus can be accessed after further dissection of the PT and exposure of the petrous apex.

**Figure 10:**
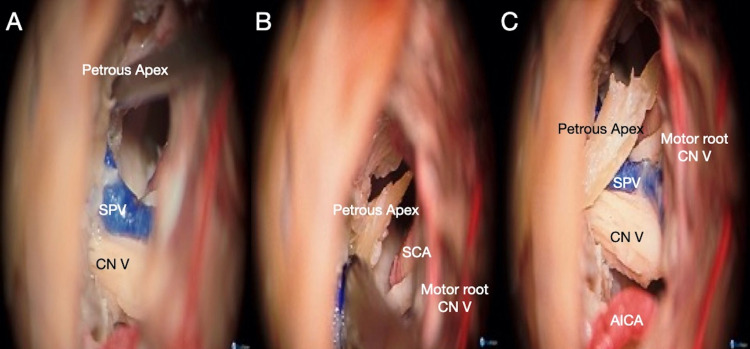
Microsurgical view of a left posterior intradural petrous apicectomy (PIPA) after removal of the suprameatal tubercle. (A) Identification and drilling of the petrous apex through the superior lateral intradural triangle with special care on the manipulation and potential head damage to adjacent neurovascular structures. (B) The petrous apex can be drilled out entirely or carefully disconnected from the petroclival junction. (C) Bone piece obtained after petrous apex removal. AICA: anterior inferior cerebellar artery; CN: cranial nerve; PIPA: posterior intradural petrous apicectomy; SCA: superior cerebellar artery; SPV: superior petrosal vein.

In the PIPA approach, access to the medial portion of the middle fossa can be achieved by identifying and removing the petrous apex. Following the removal of the suprameatal tubercle, the CN V is protected with a piece of a rubber glove, and the posterior portion of the petrous apex is removed using a diamond burr (Fig. 10) [17,18]. The removal of the petrous apex is limited superiorly by the tentorium and the SPS; anteromedially by CN VI, Dorello's Canal, and posterior cavernous sinus; inferiorly by the IPS posterolaterally by the PT (Fig 8). If further access to the middle fossa is necessary, the most anterior portion of the SPS will have to be sacrificed during the drilling of the petrous apex. It is essential to be careful about cranial nerves anteromedial to the petrous apex when opening the free edge of the tentorium (i.e., CN IV, III, and VI). The Meckel's cave and the middle fossa are properly accessed after resecting the free edge of the tentorium parallel to the petrous ridge.

Following both the RISA and PIPA procedure, the exposed petrous bone is sealed with fibrin glue, and hemostasis is achieved before closure. Thus, the closure is the same as the RS approach described above according to the respective anatomical layers.

## Conclusions

A thorough understanding of the 3D visuospatial relationship of the anatomical structures and surgical corridors in the RS approach and its intradural variations is paramount for surgical planning, successful surgeries, intraoperative decision making, and postoperative care. The RS approach can be considered the workhorse of the posterolateral cranial approaches in neurosurgery, provides a wide corridor, good working area, and angle of attack for pathologies in the posterior fossa and even extending into the cavernous sinus and the middle fossa from the posterolateral angle. This study incorporated 3D technology to create interactive VMs to facilitate a comprehensive understanding of the layer-by-layer neuroanatomical structures, major surgical techniques, and the intradural corridors of the RS approach and its variations. A precise understanding of the external myoneurovascular components is essential for avoiding postoperative complications such as skin ischemia, muscle atrophy, dysesthesia, nerve injury, and neuromas. The use of computer graphics and VR in recording and sharing neuroanatomical dissections allows for a more immersive and dynamic experience for topographic and structural studies in surgical neuroanatomy, and it can serve as valuable resources for efficient surgical workflow and anatomical education.

## References

[1] Pernczky A, Reisch R. ( 2008). Volume 1: Concept and Surgical Technique, Chapter 4: Retrosigmoid approach. Keyhole Approaches in Neurosurgery.

[2] Quiñones-Hinojosa A, Chang EF, Lawton MT (2006). The extended retrosigmoid approach: an alternative to radical cranial base approaches for posterior fossa lesions. Neurosurgery.

[3] Raza SM, Quinones-Hinojosa A (2011). The extended retrosigmoid approach for neoplastic lesions in the posterior fossa: technique modification. Neurosurg Rev.

[4] Samii M, Gerganov VM (2010). Suboccipital lateral approaches (Retrosigmoid). Cranial, Craniofacial and Skull Base Surgery.

[5] Tatagiba M, Acioly MA (2014). Retrosigmoid approach to the posterior and middle fossae.. Samii’s Essentials in Neurosurgery.

[6] Siwanuwatn R, Deshmukh P, Figueiredo EG, Crawford NR, Spetzler RF, Preul MC (2006). Quantitative analysis of the working area and angle of attack for the retrosigmoid, combined petrosal, and transcochlear approaches to the petroclival region. J Neurosurg.

[7] Rubio RR, Shehata J, Kournoutas I (2019). Construction of neuroanatomical volumetric models using 3-Dimensional scanning techniques: technical note and applications. World Neurosurg.

[8] Magill ST, Lee YM, Rodriguez Rubio R, Nguyen MP, Heilman CB, McDermott MW (2021). Retrosigmoid craniectomy with a layered soft tissue dissection and hydroxyapatite reconstruction: technical note, surgical video, regional anatomy, and outcomes. J Neurol Surg B Skull Base.

[9] Nguyen JD, Duong H (2021). Anatomy, Head and Neck, Posterior Auricular Artery. https://pubmed.ncbi.nlm.nih.gov/31536293/.

[10] Tokugawa J, Cho N, Suzuki H, Sugiyama N, Akiyama O, Nakao Y, Yamamoto T (2015). Novel classification of the posterior auricular artery based on angiographical appearance. PLoS One.

[11] Alvernia JE, Fraser K, Lanzino G (2006). The occipital artery: a microanatomical study. Neurosurgery.

[12] Louis RG Jr, Loukas M, Wartmann CT (2009). Clinical anatomy of the mastoid and occipital emissary veins in a large series. Surg Radiol Anat.

[13] Hall S, Gan Y (2019). Anatomical localization of the transverse-sigmoid sinus junction: comparison of existing techniques. Surg Neurol Int.

[14] Hall N, Sufaro Y, Kaye AH (2019). Surgical management of the cerebellopontine angle and petrous lesions. Oxford Textbook of Neurological Surgery.

[15] Kemp WJ, Cohen-Gadol AA (2011). A review of skin incisions and scalp flaps for the retromastoid approach and description of an alternative technique. Surg Neurol Int.

[16] Hoshide R, Faulkner H, Teo M, Teo C (2018). Keyhole retrosigmoid approach for large vestibular schwannomas: strategies to improve outcomes. Neurosurg Focus.

[17] Tatagiba M, Rigante L, Mesquita Filho P, Ebner FH, Roser F (2015). Endoscopic-assisted posterior intradural petrous apicectomy in petroclival meningiomas: a clinical series and assessment of perioperative morbidity. World Neurosurg.

[18] Rigante L, Herlan S, Tatagiba MS, Stanojevic M, Hirt B, Ebner FH (2016). Petrosectomy and topographical anatomy in traditional Kawase and posterior intradural petrous apicectomy (PIPA) approach: an anatomical study. World Neurosurg.

